# Role of Branched-Chain Amino Acids and Their Derivative β-Hydroxy-β-Methylbutyrate in Liver Cirrhosis

**DOI:** 10.3390/jcm11247337

**Published:** 2022-12-10

**Authors:** Silvia Espina, Alejandro Sanz-Paris, Vanesa Bernal-Monterde, Diego Casas-Deza, Jose Miguel Arbonés-Mainar

**Affiliations:** 1 Gastroenterology Department, General Hospital of Defense, 50009 Zaragoza, Spain; 2 Instituto de Investigación Sanitaria (IIS) Aragon, 50009 Zaragoza, Spain; 3 Nutrition Department, Miguel Servet University Hospital, 50009 Zaragoza, Spain; 4 Gastroenterology Department, Miguel Servet University Hospital, 50009 Zaragoza, Spain; 5 Translational Research Unit, Instituto Aragonés de Ciencias de la Salud (IACS), Miguel Servet University Hospital, 50009 Zaragoza, Spain; 6 Centro de Investigación Biomédica en Red Fisiopatología Obesidad y Nutrición (CIBERObn), Instituto Salud Carlos III, 28029 Madrid, Spain

**Keywords:** cirrhosis, nutrition, BCAA, HMB

## Abstract

Branched-chain amino acids (BCAA) supplementation is used to promote protein synthesis in different clinical conditions in which proteolysis is increased. In addition, lower plasma BCAA levels have been related to an increased risk of hepatic encephalopathy in liver cirrhosis. In this article we will review the role of supplementation with BCAAs and BCAA derivative β-hydroxy-β-methylbutyrate (HMB) in liver cirrhosis, focusing on nutritional and clinical effects. Evidence shows that BCAA supplementation slightly increases muscle mass and body mass index, with an upward trend in muscular strength and no change in fat mass. Moreover, BCAA supplementation improves symptoms of hepatic encephalopathy, and is indicated as second-line therapy. The evidence is more limited for BCAA derivatives. HMB supplementation appears to increase muscle mass in chronic diseases associated with cachexia, although this effect has not yet been clearly demonstrated in liver cirrhosis studies. To date, HMB supplementation has no clinical indication in liver cirrhosis.

## 1. Introduction

Branched-chain amino acids (BCAA) are essential nutrients whose name refers to a branched aliphatic side chain in their structure. This group of amino acids includes leucine, isoleucine, and valine [1]. Unlike most amino acids, the catabolism of dietary BCAAs is initiated in extrahepatic tissues, mainly in skeletal muscle. This gives a unique advantage to BCAA-based nutritional formulas over other supplements, especially when the target of supplementation is the muscles and brain [2]. Several studies have evaluated the effect of BCAA supplementation on conditions with high prevalence of malnutrition and sarcopenia such as old age or chronic disease, including liver cirrhosis [3]. In cirrhosis, from early stages there is an accelerated fasting metabolic disorder, with BCAAs being the main source of energy through proteolysis in skeletal muscle [4]. The beneficial effects of BCAAs in skeletal muscle are mainly due to the activation of protein synthesis via mTOR (mammalian target of rapamycin) kinase, a key regulator of protein synthesis and autophagy [3]. Accordingly, BCAA supplementation has been proposed as an alternative therapy in liver cirrhosis for the treatment of hepatic encephalopathy symptoms [5]. The objective of this article is to carry out a review of the available evidence on the nutritional and clinical effect of BCAA supplementation and its derivative β-hydroxy-β-methylbutyrate (HMB) in liver cirrhosis to clarify the indication of supplementation.

## 2. BCAA Metabolism

**Figure 1:**
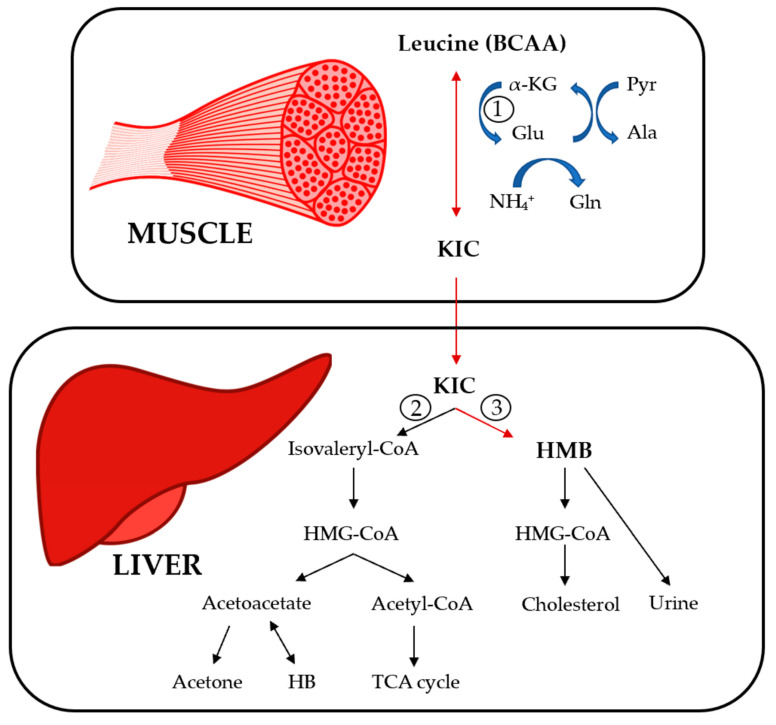
Metabolic pathway of the BCAA leucine to its derivative HMB. α-KG: α-ketoglutarate, Glu: glutamate, Pyr: pyruvate, Ala: alanine, Gln: glutamine, KIC: α-ketoisocaproate, HMB: β-hydroxy-β-methylbutyrate, HMG-CoA: 3-hydroxy-3-methyl-glutaryl-CoA, HB: beta-hydroxybutyrate.Numbers represent the key enzimes of the pathway; 1: branched-chain aminotransferase (BCAT), 2: α-keto acid dehydrogenase complex (BCKDH), 3: KIC dioxygenase.

The catabolism of BCAAs supplied in the diet begins in extrahepatic tissues, mainly in skeletal muscle, due to low activity in the liver and the high activity in skeletal muscle of the first enzyme involved in BCAA catabolism, the enzyme branched-chain aminotransferase (BCAT) [2]. The BCAT reaction involves the reversible transfer of the BCAA amino group to α-ketoglutarate to form glutamate and branched-chain keto acids (BCKAs) [2]. Glutamate acts as an amino group source to form alanine from pyruvate and as a substrate in ammonia detoxification to form glutamine [2]. The second enzyme in BCAA catabolism is the branched-chain α-keto acid dehydrogenase complex (BCKDH), which catalyzes the irreversible decarboxylation of BCKAs [2]. BCKDH activity is high in the liver, intermediate in the kidneys and heart, and low in muscle, adipose tissue and the brain [6]. BCKA metabolism leads to different metabolites such as HMB, a metabolically active derivative of leucine formed from catabolism of the BCKA α-ketoisocaproate (KIC) [7]. However, it is estimated that only 5-10% of the KIC is metabolized to produce HMB because the majority of KIC is decarboxylated by BCKDH to form isovaleryl-CoA [7]. Figure 1 summarizes the metabolic pathway of the BCAA leucine towards its derivative HMB.

## 3. Effect of BCAAs and HMB on the Regulation of Muscle Growth

### 3.1. Effect of BCAAs on Skeletal Muscle

**Figure 2:**
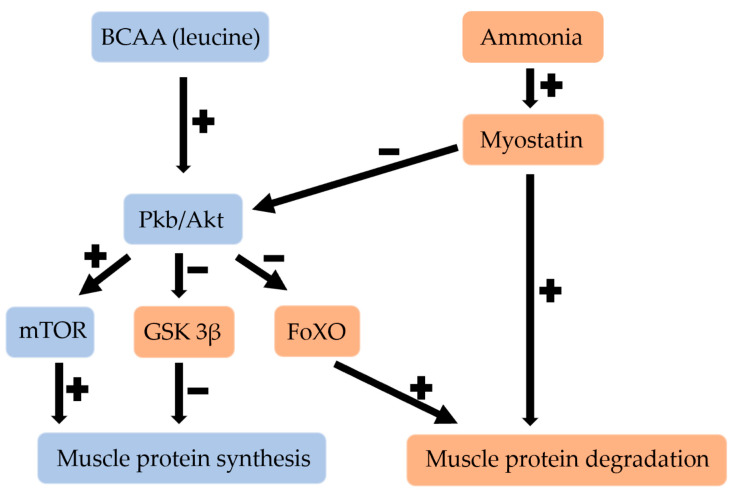
Regulation of skeletal muscle in liver cirrhosis. Blue: beneficial for skeletal muscle. Orange: harmful for skeletal muscle. BCAA: branched-chain amino acid, Pkb/Akt: protein kinase B, mTOR: mammalian target of rapamycin, GSK 3β: glycogen synthase 3 beta, FoXO: forkhead box class O. Plus and minus signs indicate up-regulation and down-regulation respectively.

BCAAs, and especially leucine, act as precursors for muscle growth. In addition, BCAAs play a role in the regulation of intracellular signals that are involved in the process of protein synthesis [8]. This is because BCAAs (leucine) activate Pkb/Akt (protein kinase B), which stimulates muscle protein synthesis through mTOR activation and GSK 3β (glycogen synthase 3 beta) inhibition. Furthermore, the activation of Pkb/Akt inhibits FoXO (forkhead box), decreasing muscle protein degradation [3]. The anabolic effect of BCAAs has only been demonstrated with leucine, and since leucine supplementation alone stimulates the catabolism of all BCAAs [8], it is proposed that BCAAs should be supplemented in the ratio 2:1:1 (Leu:Ile:Val) [9]. Conversely, myostatin, which is upregulated by ammonia, has an inhibitory effect on muscle growth through inhibition of Pkb/Akt and activating proteolysis. The main pathways for its proteolysis are the ubiquitin-proteasome pathway and the autophagy process [8]. Other factors such as exercise or testosterone also activate Pkb/Akt [8]. Figure 2 shows the regulation of skeletal muscle with BCAAs and ammonia in liver cirrhosis.

### 3.2. Effect of HMB on Skeletal Muscle

HMB is the most studied BCAA derivative. It is an active metabolic derivative of leucine that is produced in the liver from KIC. Plasma concentration of HMB increases after eating foods rich in leucine (for example, lentils, chicken, peanuts, beef, salmon, etc.) with an estimated 2–10% of leucine being oxidized to HMB [7].

**Figure 3:**
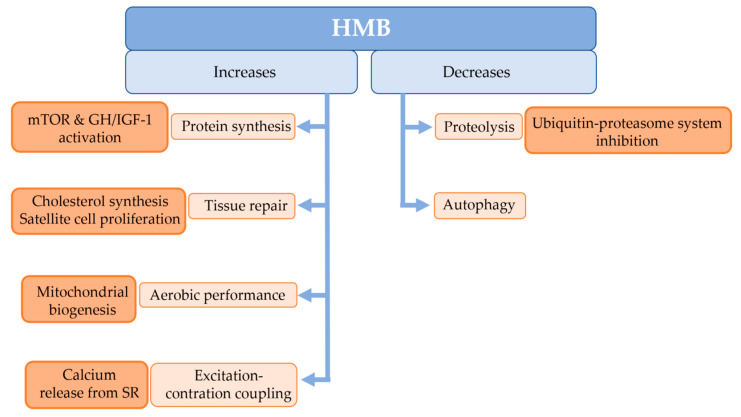
Suggested mechanisms for the effects of HMB on skeletal muscle. HMB: β-hydroxy-β-methylbutyrate, mTOR: mammalian target of rapamycin. GH/IGF-1: growth hormone/insulin-like growth factor 1, SR: sarcoplasmic reticulum.

The exogenous supply of HMB increases protein synthesis through the activation of mTOR and the GH (growth hormone)/IGF-1 (insulin-like growth factor 1) axis [7]. In addition, it has been observed in studies in vitro with muscle cells that HMB increases protein synthesis via mTOR activation more effectively than leucine [10]. Besides, HMB decreases proteolysis by inhibiting the ubiquitin-proteasome pathway and autophagy [7]. Other studies indicate that HMB increases mitochondrial biogenesis and fatty acid oxidation, increases calcium release from the sarcoplasmic reticulum, increases satellite cell proliferation and increases tissue repair through cholesterol synthesis [3]. All regulatory effects of HMB on skeletal muscle are depicted in Figure 3.

## 4. Clinical Nutrition in Liver Cirrhosis

### 4.1. Definitions and Clinical Impact of Nutrition in Liver Cirrhosis

The main disorders related to clinical nutrition are malnutrition and sarcopenia [11]. The European Society for Clinical Nutrition and Metabolism (ESPEN) defines malnutrition as “the state resulting from a lack of nutrient intake or uptake leading to altered body cell composition and mass, resulting in decreased physical and mental function”. The subtype of malnutrition that is related to chronic diseases with inflammation is synonymous with “cachexia”. In this catabolic condition, there is an inflammatory response, including anorexia and tissue breakdown, caused by an underlying disease such as cancer or liver cirrhosis [11]. The degree of metabolic response induced by the disease determines the catabolic rate and it is proportionally related to the stage of the disease [11]. ESPEN defines sarcopenia as the “syndrome characterized by a generalized loss of muscle mass, strength and function” [11]. Cirrhosis and malnutrition are generally associated with loss of skeletal muscle tissue (sarcopenia) and adipose tissue (adipopenia) [12]. Likewise, malnutrition and sarcopenia are more prevalent as liver dysfunction progresses in liver cirrhosis [13]. Child-Pugh and MELD (Model for End-Stage Liver Disease) scores have been widely used for the assessment of prognosis in liver cirrhosis according to the degree of liver dysfunction [14]. There is a higher prevalence of malnutrition and sarcopenia in patients with decompensated cirrhosis (Child-Pugh Class B or C) than in those with compensated cirrhosis (Child-Pugh Class A) [15]. In addition, all patients with decompensated cirrhosis who are candidates for liver transplantation have some degree of malnutrition [16]. Malnutrition and sarcopenia are associated with a poorer quality of life and a greater number of medical complications, such as susceptibility to infections, hepatic encephalopathy and ascites. Moreover, sarcopenia is an independent predictor of mortality beyond MELD score [17].

### 4.2. Pathogenesis of Malnutrition in Liver Cirrhosis

Protein-energy malnutrition in cirrhosis develops as a consequence of multiple factors, mainly those related to decreased caloric intake, alterations in metabolism and malabsorption. The reduction in caloric intake is mainly due to hyporexia or decreased appetite. Hyporexia in these patients has been attributed to decreased cholecystokinin clearance and increased inflammatory cytokines, such as tumor necrosis factor alpha (TNF-α), as well as hormones such as leptin [18]. Other causes of reduced caloric intake are early satiety secondary to gastric compression from ascites [19], dysgeusia, diet unpalatability, alcohol abuse or dietary restrictions secondary to long periods of hospitalization [18]. Altered macronutrient metabolism or “accelerated starvation” occurs as a result of reduced hepatic glycogen synthesis and storage, which causes a decrease in glycogen stores during the postprandial state, in turn resulting in accelerated gluconeogenesis from amino acids, which leads to muscle destruction and hyperammonemia [20]. Malabsorption causes high rates of micronutrients deficiency in patients with liver cirrhosis. Fat malabsorption is a result of intestinal edema and reduced excretion of bile salts, leading to fat-soluble vitamin deficiencies, including vitamin D. Other causes of malabsorption are bacterial overgrowth, portosystemic shunting, gastrointestinal dysmotility, pancreatic enzyme deficiency, or protein-losing enteropathy secondary to portal hypertension [18]. Deficiencies of micronutrients have been reported in patients with liver disease. Deficiency of zinc is associated with hepatic encephalopathy, frailty and sarcopenia in cirrhosis, and magnesium deficiency is associated with reduced cognitive performance and reduced muscular strength [20]. Table 1 describes the main factors involved in the pathogenesis of malnutrition in cirrhosis.

### 4.3. Nutritional Assessment in Liver Cirrhosis

The first nutritional assessment includes a measurement of weight, height, body mass index (BMI) and liver function (Child-Pugh score) [21]. Weight and therefore BMI are conditioned by the patient’s hydration status and the presence of ascites or edema. The European Association for the Study of the Liver (EASL) [22] recommends that individuals with cirrhosis and a BMI of 18.5–29.9 Kg/m² and Child-Pugh Class A or B should be evaluated using nutritional screening tools such as SGA (Subjective Global Assessment) or RFH-NPT (Royal Free Hospital Nutritional Prioritizing Tool) to screen for malnutrition. According to the degree of malnutrition, the patient is classified as low, medium or high risk of malnutrition. Patients with cirrhosis and a BMI < 18.5 kg/m² or with Child-Pugh Class C are considered to be at high risk for malnutrition. These patients with high risk for malnutrition should be seen by a nutritionist and their muscle mass evaluated to rule out sarcopenia.

The gold-standard method to evaluate sarcopenia is the computerized tomography (CT-) scan by measuring skeletal muscle cross-sectional area at the level of the third lumbar (L3), named the skeletal muscle index (SMI) [23]. Other methods to assess muscle mass are manual anthropometric measurements, muscle ultrasound, magnetic resonance imaging (MRI), dual-energy X-ray absorptiometry (DXA) or bioelectrical impedance analysis (BIA) [21]. Manual anthropometry estimates muscle mass through the measurement of circumferences, mainly mid-arm muscle circumference (MAMC), and estimates fat mass through skinfold measurement, mainly the triceps skinfold [23]. DXA and BIA measure body composition as the sum of fat mass and fat free mass. Fat free mass estimates muscle mass [24]. The evaluation of sarcopenia also includes the measurement of muscular strength, mainly with handgrip strength [25], and the measurement of muscle function with tests such as “chair stand test” [26]. When sarcopenia is present in chronic diseases such as liver cirrhosis, fat free mass and muscular strength are decreased [11]. Table 2 details the main methods to assess muscle mass and thereby also sarcopenia, as well as the most validated measurement of each method.

## 5. Metabolic Disorders in Liver Cirrhosis and Their Clinical Manifestation

The liver carries out a wide variety of functions including metabolic, vascular, immunological, secretory and excretory functions. The major metabolic functions of the liver are the regulation of the metabolism of carbohydrates, proteins and lipids [27]. In liver cirrhosis, due to impaired liver function, its metabolic functions are altered and fatty acid oxidation and gluconeogenesis are increased early in the fasting state. Thus, liver cirrhosis is considered a state of accelerated starvation. BCAAs are the main substrate for gluconeogenesis through proteolysis in the skeletal muscle [4,15]. Only BCAAs are catabolized in the skeletal muscle and as a consequence, plasma BCAA levels are lower in liver cirrhosis [4]. In contrast, the aromatic amino acids (AAA) that are also produced in the proteolysis in the skeletal muscle are primarily metabolized in the liver but, due to both hepatocellular dysfunction and portosystemic shunting, their plasma levels are increased in liver cirrhosis [4,28]. Reduced cellular amino acid concentrations activate responses that include increased skeletal muscle autophagy that has been reported in cirrhosis [4]. Fischer´s ratio is the ratio of plasma BCAA levels (leucine, valine, isoleucine) to plasma AAA levels (phenylalanine, tyrosine). As hepatic dysfunction progresses in cirrhosis, plasma BCAA levels decrease and plasma AAA levels increase, resulting in a progressive decrease in the Fischer’s ratio [28]. An inverse correlation has been observed between Fischer´s ratio and the stage of hepatic encephalopathy [29,30]. In more advanced stages of liver cirrhosis, protein synthesis is decreased [4].

In cirrhosis, there is an overproduction of ammonia and a decrease in its clearance secondary to liver dysfunction leading to hyperammonemia [31]. This hyperammonemia has been proposed as the main mediator of the liver-muscle axis [4] and its effects are well known at neurological level, producing toxicity on neurons and astrocytes [31]. Although the effect on skeletal muscle is less studied, hyperammonemia could activate the expression of myostatin, a member of the transforming growth factor-beta (TGF-β) superfamily [32,33]. Myostatin mediates the effects of hyperammonemia on the inhibition of protein synthesis and the activation of autophagy, being the link between liver dysfunction and sarcopenia [32]. Finally, when muscle mass is reduced, non-hepatic detoxification of ammonia is impaired, so when liver cirrhosis is associated with sarcopenia, complications such as hepatic encephalopathy occur more frequently; therefore, there is an inverse correlation between muscle mass and plasma ammonia [4].

## 6. Nutritional Role of BCAAs in Liver Cirrhosis

There are many studies and even meta-analyses evaluating the effect of BCAA supplementation on nutritional parameters in liver cirrhosis. However, the discrepancies in the results are conditioned by the heterogeneity of the selected population, by heterogeneity in the type of BCAA supplementation, and by heterogeneity in the measures analyzed. Regarding the heterogeneity of the selected population, liver cirrhosis can be compensated or decompensated and may or may not include conditions that affect malnutrition such as refractory ascites, hepatocarcinoma, associated sarcopenia, or hospitalization, among others. Regarding the heterogeneity in the type of supplementation, BCAAs can be administered in granules, dissolved in water or other hypocaloric substances (for example, juice), or be administered under formulas of hypercaloric nutrients as oral nutritional supplementation (ONS) (mainly, Aminoleban^®^, which provides 210 Kcal per unit) [34]. Obviously, BCAA supplementation under hypercaloric nutrients provides a higher daily caloric intake than BCAA supplementation in granules. Additionally, it is also worth differentiating between studies that supplement BCAAs for three to six months (short duration) versus studies lasting longer than six months (long duration). Finally, it should be noted that the control group can be placebo (casein) or diet, but also a standard ONS regimen that usually contains leucine and consequently reduced doses of BCAAs. Therefore, the choice of the control group greatly influences the results obtained. Lastly, regarding the heterogeneity of the measures analyzed, some anthropometric methods are influenced by fluctuations in hydration such as manual anthropometry, BIA or DXA [21]. The estimation of muscle mass by measuring the SMI by CT is the gold-standard [23], compared to other methods such as the measurement of MAMC by manual anthropometry or the fat free mass by BIA or DXA. For all these reasons, it is difficult to reach firm conclusions regarding the results of BCAA supplementation on nutritional parameters in individuals with cirrhosis.

### 6.1. Effect on Body Composition and Muscular Strength

BCAA supplementation is one of the therapies for the treatment of sarcopenia in liver cirrhosis [22], based on its effect on muscle protein growth [3,33]. The most recent bibliography is presented, which is a review of the previous literature.

Ooi et al. [35] reviewed the effect of BCAA supplementation as part of hypo- or hyper-caloric formulas on anthropometry in individuals with liver cirrhosis, including a total of 40 studies, and evaluating their effect before and after BCAA supplementation. The authors concluded that most of the studies (76%) did not observe changes in weight. Regarding body composition, in the majority (75%) no significance was observed in the variation of fat mass or in the estimation of muscle mass; and in terms of muscular strength, 75% of them found a positive effect with an increase of 4% at the end of supplementation. Ismaiel et al. [36] have recently published a meta-analysis of 17 studies evaluating the effect of BCAA supplementation versus other therapies (control) on parameters that measure sarcopenia in liver cirrhosis. The studies included supplementary BCAAs under hypo- or hyper-caloric formulas. I² statistic was used for quantification of heterogeneity. The results on the estimation of muscle mass found a significant increase in SMI (I² = 0%) and in MAMC (I² = 90.98%) at the end of BCAA supplementation, with no differences in MAMC post-intervention compared to control therapy (I² = 75.63%). Regarding muscular strength, evaluated with handgrip strength, a non-significant increase was observed at the end of BCAA supplementation (I² = 59.29%) and compared to control. Regarding fat mass, measured by the triceps skinfold, a non-significant decrease was observed at the end of therapy with BCAA supplementation compared to control. The most recent meta-analysis about BCAA supplementation in liver cirrhosis, by Konstantis et al. [37], included 20 randomized clinical trials with oral BCAA supplementation, as part of hypo- or hyper-caloric formulas. Compared to the control group, they observed a slight but significant difference in muscle mass estimated with SMI and/or MAMC in favor of the BCAA group (I² = 0%). No significant differences were observed between groups (BCAA vs control) in the variation of fat mass measured via triceps skinfold, although in the BCAA group the trend was downward (I² = 0%). Lastly, the BMI did increase significantly in the group of patients supplemented with BCAAs compared to the control group (I² = 0%). The results seem to indicate that BCAA supplementation slightly increases muscle mass and function in patients with liver cirrhosis. In addition, it seems that the effect of BCAAs on muscle mass is greater in those with sarcopenia at baseline [37,38]. The results on body composition of the previously cited meta-analysis and systematic review are summarized in Table 3.

### 6.2. Effect on Serum Albumin

Albumin is a protein synthesized in the liver. Its plasma levels have been traditionally used to monitor long-term nutritional therapies due to its long half-life [39]. However, albumin also reflects liver function and its levels are decreased in advanced liver cirrhosis, with or without malnutrition [21].

Regarding the effect on serum albumin, Konstantis et al. [37] observed a significant increase in albumin levels with BCAA supplementation compared to other therapies in patients with liver cirrhosis, more evident with more severe hypoalbuminemia at baseline and a longer supplementation time (>12 weeks), although the results were heterogeneous (I² = 84.99%). Ishikawa et al. [40] observed that in early stages of liver cirrhosis, with serum albumin between 3.5 and 3.9 g/dL, the effect of BCAA supplementation is greater when the BCAA/tyrosine ratio is <4. Furthermore, it seems that BCAA supplementation in early stages of cirrhosis had a greater effect on serum albumin than in more advanced stages [35,41].

## 7. Clinical Role of BCAAs in Liver Cirrhosis

### 7.1. Effect on Complications of Liver Cirrhosis

The Cochrane systematic review carried out in 2012 [42] evaluated the effect of different nutritional therapies in liver cirrhosis, establishing that patients with liver cirrhosis who received oral nutritional supplements (ONS) had a lower incidence of ascites, possibly a lower incidence of infections and a faster resolution of hepatic encephalopathy symptoms. However, the improvement in hepatic encephalopathy symptoms only occurred in the BCAA-supplemented group. The Cochrane systematic review found no improvement in prevention of hepatic encephalopathy or resolution of ascites with ONS [42]. Ascites is part of the pathogenesis of malnutrition because it produces gastric compression that leads to a decrease in nutrient intake due to early satiety [18,19]. ONS supplementation in liver cirrhosis, based on regimens with or without BCAAs, decreases the incidence of ascites but does not resolve the problem [42]. In 2017, the Cochrane database [5] conducted another systematic review of the effects of BCAA supplementation on liver cirrhosis with hepatic encephalopathy, both minimal and overt. The authors found that BCAA supplementation produced a significant improvement in the symptoms of hepatic encephalopathy, and no difference was found between BCAA supplementation and lactulose or neomycin therapy in improving hepatic encephalopathy symptoms. Konstantis et al. [37] found that the BCAA-supplemented group reduced the incidence of serious cirrhotic complications (I² = 0%) such as development of ascites, hepatocellular carcinoma, varices rupture, hepatic encephalopathy and serious infections.

**Figure 4:**
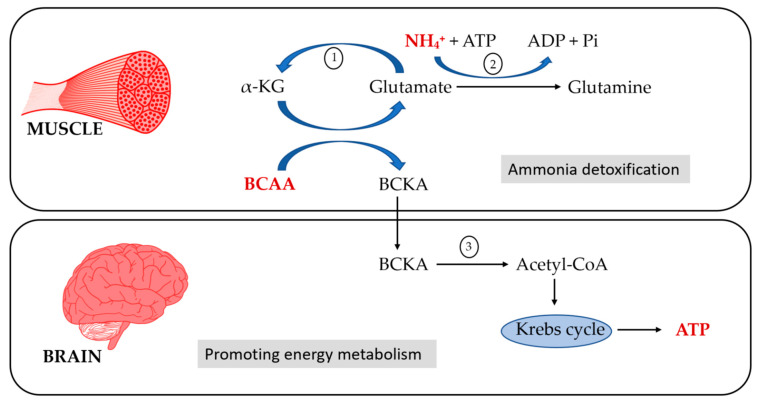
Pathophysiological mechanisms by which BCAA supplementation improves hepatic encephalopathy symptoms. BCAA: branched-chain amino-acid, BCKA: branched-chain keto acid, α-KG: α-ketoglutarate. Numbers represent key enzimes of the process; 1: glutamate dehydrogenase, 2: glutamine synthetase, 3: ranched-chain α-keto acid dehydrogenase complex.

The possible pathophysiological mechanisms by which BCAA supplementation improved symptoms of hepatic encephalopathy could be the following: (1) BCAAs may increase ammonia detoxification via α-ketoglutarate in skeletal muscle, lowering hyperammonemia [9]; (2) BCAAs could serve as an anaplerotic substrate for the brain, improving its energy metabolism; and (3) BCAAs might reduce the cerebral flow of AAA [43]. Figure 4 represents the mechanisms by which BCAAs would improve the symptoms of hepatic encephalopathy.

### 7.2. Effect on Mortality and Quality of Life

Malnutrition and sarcopenia in liver cirrhosis are associated with lower quality of life and a greater number of medical complications. Furthermore, sarcopenia is an independent predictor of lower survival in liver cirrhosis and in patients waiting for liver transplant [22]. The meta-analysis published by Konstantis et al. [37] of the effect of supplementation in liver cirrhosis found no differences on mortality between BCAA supplementation versus other therapies. The Cochrane systematic review in 2012 [42] found no difference on mortality with any type of supplementation in liver cirrhosis, and the latest Cochrane systematic review in 2017 [5] noted that BCAA supplementation in liver cirrhosis with hepatic encephalopathy did not impact on mortality or quality of life. However, some improvement in quality of life was observed with long-term BCAA supplementation [44,45]. Muto et al. [45] observed that long-term BCAA supplementation for 2 years decreased mortality due to liver decompensation events.

## 8. Recommendations of the Main Scientific Societies Regarding BCAA Supplementation in Liver Cirrhosis

The growing literature on the nutritional and clinical effect of BCAA supplementation in liver cirrhosis and the knowledge of the impact of the nutritional status in the prognosis of these patients has led the main scientific societies to establish their nutritional recommendations, both general and specific on supplementation with BCAAs.

According to the EASL, in the latest guideline on nutrition in chronic liver disease published in 2019 [22]:

- Patients with liver cirrhosis who present malnutrition and/or sarcopenia with BMI < 30 kg/m² who do not reach the recommended nutritional intake of 30–35 Kcal/Kg/day, nutritional supplementation can be chosen, preferably oral, and can be with a standard amino acid regimen or based on BCAAs.
- Patients with liver cirrhosis who do not reach a protein intake of 1.2 g/Kg/day, or 1.5 g/Kg/day if they have sarcopenia, or have intolerance to animal proteins, can opt for replacement with vegetable proteins and BCAA supplementation.
- Patients with liver cirrhosis and hepatic encephalopathy can be considered for oral BCAA supplementation to improve neuropsychiatric performance and to reach the recommended nitrogen intake, but their use intravenously for episodic overt hepatic encephalopathy is not supported.

According to the ESPEN, in the latest recommendations published in 2020 [46] on clinical nutrition in liver cirrhosis:

- Patients with liver cirrhosis and intolerance to animal proteins, vegetable proteins or oral BCAA (0.25 g/kg/day) supplementation should be used to facilitate adequate protein intake.
- Long-term BCAA (0.25 g/kg/day) supplementation in advanced-stage liver cirrhosis may be prescribed to improve survival and quality of life.
- After surgery or in the pre-liver transplant period, BCAA-based regimens have not been superior to other standard regimens with respect to morbidity and mortality.

According to the American Association for the Study of Liver Diseases (AASLD) [20], in the latest guideline on malnutrition, frailty and sarcopenia in patients with cirrhosis published in 2021:

- BCAA supplementation is not recommended beyond emphasizing the importance of meeting daily overall protein targets from a diverse range of protein sources.

The practice guideline by the ESPEN and AASLD on management of hepatic encephalopathy in chronic liver disease published in 2014 [47] established that oral BCAA supplementation can be used as an alternative or additional agent to treat patients nonresponsive to conventional therapy (Grade I, B, 2).

## 9. Effect of HMB in Liver Cirrhosis

### 9.1. Nutritional Role of HMB in Liver Cirrhosis

It has been observed that there is an increased flow of α-ketoisocaproate toward the enzyme branched-chain α-keto acid dehydrogenase complex in liver cirrhosis, conditioning a decrease in the production of HMB [7].

A recent meta-analysis published by Bear et al. [48] on the effects of HMB supplementation in patients with cachexia, which included 15 randomized clinical trials, showed an increase in skeletal muscle mass and muscular strength, although the effect size was small, with no change in body weight. HMB was supplemented either alone, associated with hypocaloric nutrients or under ONS.

The literature about the effects of HMB on liver cirrhosis to date only includes two clinical trials and one clinical trial after liver transplantation. Lattanzi et al. [49] studied the effect of HMB after liver transplantation. Patients supplemented with 3g daily of HMB dissolved in juice for 12 weeks saw an increase in muscle mass at the end of supplementation and maintained this at 12 months compared to placebo. Muscle mass was estimated with anthropometry using the MAMC measure and with the appendix skeletal muscle mass index (ASMI) measured by DXA. An increase in muscular strength estimated with hand grip strength was only observed in the HMB group at the end of supplementation and maintained at 12 months. The same group later published [50] the results of a clinical trial in compensated liver cirrhosis (90% Child-Pugh class A) after 12 weeks of supplementation with 3 g daily of HMB dissolved in water versus placebo. The results observed at the end of supplementation were a statistically significant increase of quadriceps muscle mass measured by ultrasound and in muscle function tests in the HMB group. However, no significant improvement was found in the HMB group versus placebo in the parameters of muscle mass estimated by BIA or in muscular strength. Our group has recently published [51] a clinical trial of the effect of an ONS enriched with 1.5 g of HMB given twice daily for 12 weeks to patients with liver cirrhosis and at least one previous liver decompensation. A control group received ONS twice a day with a similar composition but without HMB. We observed a statistically significant increase in weight and fat mass at the end of supplementation in the HMB group, without differences with the control group. No variation in muscle mass estimated by BIA and manual anthropometry was observed in any of the groups. Only in the HMB group was an upward trend observed in muscular strength estimated with hand grip strength. Table 4 summarizes the aforementioned clinical trials of the effect of HMB on body composition.

### 9.2. Clinical Role of HMB in Liver Cirrhosis

The literature on the clinical role of HMB supplementation in cirrhosis is scarce. Only the results of the clinical trial in compensated liver cirrhosis published by Lattanzi et al. [50] and the clinical trial in decompensated liver cirrhosis published by Espina et al. [51] are available. The former [50] did not show a change in the score of the psychometric tests that evaluate minimal hepatic encephalopathy (MHE), although the population included did not have MHE at baseline. There were also no variations in the score of the prognostic scales of liver cirrhosis (MELD and Child-Pugh score). The latter [51] showed an HMB-specific reduction of patients with MHE, not observed in the control group. Furthermore, the presence of MHE was also correlated with reduced BCAA plasma levels and a lower Fischer ratio [30]. HMB supplementation did not improve prognostic scales of liver cirrhosis (MELD and Child-Pugh score), prevent decompensation events, or decrease mortality compared to the control group [51].

## 10. Conclusions

BCAA supplementation in patients with liver cirrhosis and malnutrition and/or sarcopenia appears to slightly increase muscle mass and function. Additionally, BCAA supplementation seems to improve symptoms of hepatic encephalopathy by increasing ammonia detoxification. However, the benefits of BCAA supplementation on prognosis and quality of life are still unclear. The use of BCAA carries a significant economic cost, and so consequently the indications should be well established. BCAA supplementation in liver cirrhosis is indicated when (1) intolerance or ethical issues with animal proteins are present in patients with malnutrition or sarcopenia, (2) as an ONS option when malnutrition and/or sarcopenia is present and patients do not reach the recommended nutritional intake, or (3) as second-line therapy in hepatic encephalopathy when standard therapy fails. The leucine derivative HMB has been used as a treatment for sarcopenia in chronic diseases similar to cirrhosis, but there is currently no firm evidence for the indication or specific recommendation for HMB supplementation in liver cirrhosis. More homogeneous meta-analyses of the effect of BCAA supplementation on body composition in patients with liver cirrhosis are needed. The effect of BCAA and/or HMB supplementation on long-term prognosis and quality of life should also be clarified. Future lines of research on the effect of HMB on liver cirrhosis and sarcopenia, and its clinical impact, are suggested.

## Figures and Tables

**Table 1:** Pathogenesis of malnutrition in cirrhosis.

Decreased Caloric Intake	Malabsorption	Altered Metabolism
Hyporexia	Altered enterohepatic circulation of bile salts	Reduced glycogen synthesis
Early satiety	Bacterial overgrowth	Increased gluconeogenesis
Dysgeusia	Portosystemic shunting	Increased fatty acid oxidation
Diet unpalatability	Gastrointestinal dysmotility	Protein and fat breakdown
Alcohol abuse	Pancreatic enzyme deficiency	
Dietary restrictions	Enteropathy	

**Table 2:** Main methods and measurements to assess sarcopenia.

Sarcopenia	
Method	Measurement
Manual anthropometry	MAMC
Ultrasound	Muscle thickness
BIA	Fat free mass
DXA	Fat free mass
CT	L3 SMI
Muscular strength	Handgrip strength

MAMC: mid-arm muscle circumference, BIA: bioelectrical impedance analysis, DXA: dual energy X-ray absorptiometry, CT: computerized tomography, SMI: skeletal muscle index.

**Table 3:** Characteristics of the main review articles on BCAA supplementation in cirrhosis.

Study, Year	Study Type	Number of Studies Included	Population	BCAA Intervention	Comparison	Measurements	Main Findings
Konstantis et al., 2022 [37]	Meta-analysis	20	Adults with cirrhosis, including HCC	Hypo- or hyper- caloric formulas	Diet, snacks, M-DXT, L-ALB & casein	SMI and/or MAMC	Slight significant increase in BCAA group compared to control group
						Tricipital skinfold	Decreasing trend in the BCAA group, without differences with the control
						BMI	Significant increase in the BCAA compared to control
Ismaiel et al., 2022 [36]	Meta-analysis	17	Adults with cirrhosis, excluding HCC	Hypo- or hyper- caloric formulas	M-DXT, diet, L-ALB & No group of comparison	SMI and/or MAMC	Significant increase in BCAA group, without differences compared to control
						Handgrip strength	Non-significant increase at the end of BCAA therapy and compared to control
						Tricipital skinfold	Non-significant decrease with BCAA vs control therapy
Ooi et al., 2018 [35]	Systematic review	40	Children & adults with cirrhosis or hepatic failure, including HCC	Hypo- or hyper- caloric formulas	Diet, casein, etc & No group of comparison	Fat free mass	No variation in 75% of studies
						Muscular strength	Increased in 75% of studies
						Fat mass	No variation in 75% of studies
						Body weight	No variation in 76% of studies

HCC: hepatocellular carcinoma, M-DXT; maltodextrins, L-ALB: lactalbumin, SMI: skeletal muscle index, MAMC: mid-arm muscle circumference, BMI: body mass index.

**Table 4:** Characteristics of the main clinical trials on HMB supplementation in cirrhosis.

Study, Year	Study Type	Population	HMB Intervention	Comparison	Measurements	Main Findings
Lattanzi et al., 2019 [49]	Clinical trial	After liver transplantation (n = 22)	3g daily of HMB dissolved in juice	Placebo	DXA (ASMI), anthropometry (MAMC) and hand grip strength	All increases only in HMB group
Lattanzi et al., 2021 [50]	Clinical trial	Compensated liver cirrhosis (n = 24)	3g daily of HMB dissolved in water	Placebo	Quadriceps ultrasound, muscle function tests, BIA, and hand grip strength	Increase in the HMB group of quadriceps muscle mass and muscle function tests No variation in BIA parameters or in hand grip strength
Espina et al., 2022 [51]	Clinical trial	Decompensated liver cirrhosis (n = 43)	ONS with 1.5g of HMB, twice a day	ONS without HMB, twice a day	BIA (FM, FFM), anthropometry (weight, MAMC), and hand grip strength	Increase in both groups of weight and FM No variation in FFM and MAMC Upward trend in hand grip strength in HMB group

HMB: β-hydroxy-β-methylbutyrate, DXA: dual-energy X-ray absorptiometry, ASMI: appendix skeletal muscle mass index, MAMC: mid-arm muscle circumference, BIA: bioelectric impedance analysis, ONS: oral nutritional supplement, FM: fat mass, FFM: fat free mass.

## Data Availability

Not applicable.
